# Impact of intravesical Bacillus Calmette-Guérin and chemotherapy on the bladder microbiome in patients with non-muscle invasive bladder cancer

**DOI:** 10.3389/fcimb.2023.1125809

**Published:** 2023-04-05

**Authors:** Christopher James, Kayeromi Gomez, Shalin Desai, Hiten D. Patel, Goran Rac, Chirag P. Doshi, Ryan Dornbier, Petar Bajic, Thomas Halverson, Gopal N. Gupta, Marcus L. Quek, Alex Gorbonos, Robert Flanigan, Alan J. Wolfe

**Affiliations:** ^1^ Loyola University Medical Center, Department of Urology, Maywood, IL, United States; ^2^ Department of Hematology/Oncology, Loyola University Chicago, Stritch School of Medicine Maywood, IL, United States; ^3^ Department of Urology, Feinberg School of Medicine, Northwestern University, Chicago, IL, United States; ^4^ Department of Urology, Cleveland Clinic Lerner College of Medicine, Cleveland, OH, United States; ^5^ Loyola University Chicago, Department of Microbiology and Immunology, Maywood, IL, United States

**Keywords:** Bacillus Calmette-Guérin (BCG), intravesical therapy, microbiome, bladder cancer, intravesical gemcitabine

## Abstract

**Introduction:**

Intravesical therapy (IVT), including Bacillus Calmette-Guérin (BCG), is the standard of care for high grade (HG) non-muscle invasive bladder cancer (NMIBC). Despite the use of IVT, many patients recur after treatment. The bladder microbiome and its role in disease processes has recently risen to prominence. We aim to characterize changes that occur in the bladder microbiome over the course of intravesical therapy and assess whether these changes correlate with outcomes in patients with NMIBC.

**Methods:**

Patients with NMIBC undergoing induction BCG or intravesical therapy were prospectively enrolled from January 2019 to March 2020. Patients with clinical T2 or greater pathology or active urinary tract infection at enrollment were excluded. Twenty-nine patients had catheterized (bladder) urine samples collected prior to induction intravesical therapy and prior to each IVT instillation. Twenty-seven received BCG while 2 received intravesical gemcitabine. Bacteria were identified using 16S ribosomal RNA gene sequencing. Bladder microbiome changes were evaluated and differences between patients who recurred and patients who did not recur after IVT were investigated.

**Results:**

Across the 29 patients analyzed, bacterial richness decreased significantly following intravesical therapy (Richness, P=0.01). Evenness and overall diversity did not change significantly (Pielou, P=0.62; Shannon, P=0.13). Patients who experienced recurrence had a higher relative abundance of *Aerococcus* in their urine (P<0.01), while those who did not recur had significantly more *Ureaplasma* (P=0.01) and *Escherichia/Shigella* species (P=0.05). Patients with decreased levels of alpha diversity were more likely to fall within the non-recurrence cohort.

**Conclusion:**

IVT for NMIBC appears to change the urinary microbiome by decreasing richness while not altering evenness or overall diversity. The presence of *Aerococcus* species may be predictive of a poor cancer response to IVT, while the presence of *Ureaplasma* and *Escherichia/Shigella* may predict a favorable response to IVT. Further studies are warranted to elucidate and confirm the significance of changes in the bladder microbiome.

## Introduction

Bladder cancer is the fourth most common cancer in men and fifth most common cancer overall (Ploeg et al., 2009). With 83,730 new cases of bladder cancer reported in 2021 and over 17,000 deaths from bladder cancer expected in the United States (Siegel et al., 2021), treating this malignancy remains a challenge for urologists as many of the mainstay treatment therapies and surgeries carry high morbidity.

The internal surface of the bladder is lined by an impermeable transitional cell epithelium known as the urothelium (Lewis, 2000). The most common type of bladder cancer, urothelial carcinoma, arises from this cell layer.

For intermediate- and high-risk urothelial bladder cancers that have not invaded the muscular layer, non-muscle invasive bladder cancer (NMIBC), the treatment mainstay is maximal endoscopic tumor resection followed by intravesical therapy (Lamm et al., 2000). Of the intravesical therapies available, Bacillus Calmette-Guerin (BCG) is the most widely used (American Cancer Society, 2022).

While BCG clearly causes tumor regression in many patients, it is not always effective. Although many would argue that the exact mechanism by which BCG treats NMIBC is not fully understood, it is widely recognized that BCG stimulates the immune system. Preliminary studies show the degree of immune response, assessed by urinary cytokine quantification, is associated with rates of recurrence and progression (Redelman-Sidi et al., 2014). However, current models do not account for the recent discovery of resident microbial communities (microbiota) in the bladder.

Gemcitabine has also been approved for use as adjuvant intravesical therapy for NMIBC. Gemcitabine is a nucleoside analog that masquerades as cytidine and incorporates into replicating DNA leading to chain termination (Skinner, 2013). Emerging data show interplay between the gut microbiome and systemic chemotherapeutic agents with regards to treatment efficacy and toxicity (Alexander et al., 2017). However, to the best of our knowledge, no one has investigated the bladder microbiome with regards to intravesical chemotherapeutic agents such as gemcitabine.

Given these considerations, we contend that the relationship between intravesical therapy, host immunity, and the bladder microbiome warrants study. In this investigation, we sought to evaluate changes to the bladder microbiome at various timepoints throughout induction intravesical therapy. We also aimed to determine associations between the bladder microbiome and intravesical therapy efficacy. A greater understanding of the interplay between the bladder microbiome and intravesical therapies may guide future directions in bladder cancer diagnostics and treatment.

## Methods

### Study design and patient population

Following Institutional Review Board (IRB) approval (LU211552) at Loyola University Medical Center, we enrolled 29 participants of any age and gender with recently diagnosed high-grade NMIBC on transurethral resection of bladder tumor (TURBT), who were to undergo an induction course intravesical therapy with either BCG or gemcitabine. Patients with muscle-invasive bladder cancer, low-grade bladder cancer, prior intravesical therapy, active UTI at time of first intravesical therapy and those pregnant at time of treatment were excluded. One patient within the cohort received an intraoperative dose of gemcitabine at the time of TURBT and then went on to receive induction BCG. Participants received cancer care at Loyola University Medical Center in Maywood, IL between January 2019 and March 2020. All participants signed a written research consent giving permission for extraction of baseline demographic and clinical data from their electronic medical record. Baseline demographics are shown in **Table 1**.

**Table 1:** Participant demographics.

	Overall
Demographic Variable	Total N=29
Age at Diagnosis, ~ mean (SD)	67.8 (11.7)
Race	
White	21 (72.4)
Black	5 (17.2)
Hispanic	1 (3.5)
Other	2 (6.9)
Sex	
Female	7 (24.1)
Male	22 (75.9)
Smoking History	
Yes	20 (69)
No	9 (31)
Family History of UCC	
Yes	2 (6.9)
No	27 (93.1)
History of High Grade UCC	
Yes	0 (0)
No	29 (100)
History of Low Grade	
Yes	3 (10.3)
No	26 (89.7)
History of Upper Tract	
Yes	2 (6.9)
No	27 (93.1)
Prior Cancer History	
Some History	5 (17.2)
No prior History	24 (82.8)

Unless otherwise indicated, all summaries are recorded as Count (%).

UCC, urothelial cell carcinoma.

SD, Standard Deviation.

### Sample collection and analysis

Enrolled participants were catheterized aseptically for a urine specimen prior to their first instillation of induction intravesical therapy. Subsequent aseptic catheterized specimens were obtained prior to all 6 weekly treatments as part of each patient’s induction course. A 7^th^ catheterized specimen was obtained prior to first follow-up cystoscopy, which was performed 3 months after completion of induction intravesical therapy course. Each urine specimen was transported to the Wolfe Lab (Maywood, IL, USA) within 4 hours of collection for analysis.

### 16S rRNA gene sequencing and bioinformatics analysis

An aliquot of urine from each timepoint was preserved in 10% AssayAssure and stored at -80°C for future, batched 16S rRNA gene sequencing. The protocol for DNA extraction and isolation, polymerase chain reaction (PCR) amplification and 16S rRNA gene sequencing of urine samples has been described previously (Fok et al., 2018).

First, we extracted and isolated genomic DNA from each 1 ml urine specimen in a PCR hood using previously validated protocols (Hilt et al., 2014; Pearce et al., 2014; Price et al., 2016)This protocol includes the peptidoglycan degrading enzymes mutanolysin and lysozyme to promote sufficient lysis of both gram-positive and gram-negative bacteria (Yuan et al., 2012). Briefly, urine specimens were centrifuged at 13,500 rpm for 10 minutes. We then resuspended the pellet in 200 microliters of filter-sterilized buffer consisting of 20 mM Tris-Cl (pH 8), 2mM EDTA, 1.2% Triton X-100, and 20 micrograms/ml lysozyme supplemented with 20 microliters of filter-sterilized mutanolysin (5,000 U/ml; Sigma-Aldrich, St. Louis, MO). We incubated the mixture at 37°C for 1 hour and processed the lysates with the DNeasy blood and tissue kit (Qiagen, Valencia, CA), according to the manufacturer’s protocol. Genomic DNA was eluted into 50 μL of buffer AE (pH 8.0) and stored at -20°C.

Using the stored genomic DNA as a template, we amplified the hyper-variable region 4 (V4) of the bacterial 16S rRNA gene with a two-step PCR protocol, as described previously (Hilt et al., 2014; Pearce et al., 2014). We first amplified the V4 region using Illumina MiSeq modified universal primers 515F and 806R. We included extraction negative controls (no urine or swab suspension) to identify contaminating DNA from reagents. We also included PCR-negative controls to monitor for any potential cross-contamination that occurred during the nucleic acid extraction process. We diluted the reaction mixtures 1:50 and amplified them for 10 more cycles, using primers that include the adapter sequences for Illumina MiSeq sequencing and an 8-nucleotide sample index. We purified the PCR reaction and then size selected using Agencourt AMPure XP-PCR magnetic beads (Beckman Coulter, Pasadena, CA). We quantified each sample with the Qubit fluorometric system (Thermo-Fisher, Waltham, MA). We combined the samples into a common batch, normalized to a standard volume, and inserted them in the 2 x 250 bp sequencing reagent cartridge, according to manufacturer protocol instructions (Illumina, San Diego, CA).

We then quality trimmed the raw reads using Cutadapt (v.2.7) (Martin, 2011) to remove adaptors from both ends and processed the trimmed reads using the DADA2 package (v.1.14.1) in R (v.3.6) (Callahan et al., 2016). This function included quality control, error rate calculation, dereplication, and chimera removal to generate amplicon sequence variant (ASV) tables. We used the SILVA database (v.132) (Quast et al., 2013) to assign taxonomy to the sequences in the ASV table. Negative and extraction controls (n=14) were used to identify potential contaminants using the decontam package (v1.7) (Davis et al., 2018). To ensure we analyzed only ASVs that were confidently not contaminants, we manually removed ASVs whose reads did not exceed 5 times the maximum number of reads in extraction and negative controls. Samples with <1000 reads were discarded from downstream analysis, as removal of suspected contaminants from these samples substantially altered alpha diversity. After decontamination, we obtained taxonomic identities for the remaining ASVs using BLCA (github.com/qunfengdong/BLCA), which can often achieve species-level identification (Gao et al., 2017).

### Statistical analyses

Descriptive statistics for baseline demographics and clinical characteristics were calculated. Comparisons between recurrence and non-recurrence group were carried using Wilcoxon rank-sum tests for continuous variables and Fisher exact tests for categorical variables. Kruskal Wallis tests were also used for comparison across the three timepoints namely the beginning, the middle and the end. Medians and interquartile ranges (IQRs) were used to summarize the diversity measures overall, for those who recur and those who did not as well as for patients in the three timepoints groups. The relative abundance of each species was computed and recorded as mean (standard deviation) and bacterial compositions were compared using four measures of alpha diversity. Richness was calculated using the number (counts) of unique taxa. The distribution of bacterial taxa within samples (evenness) was computed with the Pielou index. Combined interactions were calculated with the Shannon index (richness and evenness) and Simpson index (richness and taxon abundance). All these calculations as well as all data analyses were performed using the SAS statistical software (version 9.4; SAS Institute Inc, Cary, NC).

## Results

We assessed the bladder microbiome of 29 patients undergoing intravesical therapy for NMIBC at various timepoints in their treatment course. Twenty-seven patients received BCG and two patients received intravesical gemcitabine. Catheterized urine samples were obtained prior to initiation of intravesical therapy, before each intravesical therapy treatment, and after conclusion of intravesical therapy treatment.

**Tables 1**, **2** show the basic demographic data across the entire cohort and between non-recurrence and recurrence groups, respectively. For our cohort, the mean age at diagnosis was 67.8 (SD 11.7) and most patients were Caucasian (n=21, 72.4%). The remainder were Black (n=5, 17.2%), Hispanic (n=1, 3.5%) and other race including Asian, mixed race and American Indian (n=2, 6.9%). The cohort was comprised of 22 males (75.9%) and 7 females (24.1%). Twenty patients reported a history of smoking (69%) and 2 had a family history of urothelial cell carcinoma (6.9%). No patients had a personal history of high-grade urothelial cell carcinoma of the bladder; however, 3 patients had a history of low-grade urothelial cell carcinoma of the bladder (10.3%) and 2 patients had a history of upper tract urothelial carcinoma (6.9%).

**Table 2:** Participants characteristics (with recurrence status).

	Recurrence		
	Yes (N=8)	No (N=21)	P-value
Age at Diagnosis, ~ mean (SD)	66 (10.3)	68.5 (12.4)	0.79^w^
Race			
White	8 (100)	13 (61.9)	
Black	0 (0)	5 (23.8)	
Hispanic	0 (0)	1 (4.8)	
Other	0 (0)	2 (9.5)	<0.01^f^
Sex			
Female	2 (25)	5 (23.8)	
Male	6 (75)	16 (76.2)	1.00^f^
Smoking History			
Yes	4 (50)	16 (76.2)	
No	4(50)	5 (23.8)	0.21^f^
Family History of UCC			
Yes	1 (12.5)	1 (4.8)	
No	7 (87.5)	20 (95.2)	0.48^f^
History of High Grade UCC			
Yes	0 (0)	0 (0)	
No	8 (100)	21 (100)	<0.01^f^
History of Low Grade			
Yes	2 (25)	1 (4.8)	
No	6 (75)	20 (95.2)	0.18^f^
History of Upper Tract			
Yes	1 (12.5)	1 (4.8)	
No	7 (87.5)	20 (95.2)	0.48^f^
Prior Cancer History			
Some History	3 (37.5))	2 (9.5)	
No prior History	5 (62.5)	19 (90.5)	0.11^f^

Unless otherwise indicated, all summaries are recorded as Count (%).

UCC, urothelial cell carcinoma.

SD, Standard Deviation.

P-values with f represent Fisher Exact Test.

P-values with w represent Wilcoxon Rank Sum Test.

We analyzed the composition of the bladder microbiome 2 different ways: for the entire cohort over time and between patients who experienced recurrence of bladder cancer following intravesical therapy versus patients who did not recur. **Supplemental Figure 1** shows the prevalence of genera detected in our entire cohort by 16S rRNA sequencing across three timepoints relative to treatment: beginning, middle and end. Beginning timepoints included those samples obtained before initiation of intravesical therapy. Middle timepoints included samples obtained after at least one instillation of intravesical therapy but prior to last instillation of intravesical therapy. End timepoints included samples obtained prior to final instillation of intravesical therapy or after all intravesical therapy treatments were completed. Two observations are apparent. First, we were able to detect *Mycobacterium*, the genus of the BCG bacterium. Second, the most prevalent genera did not change dramatically over time. **Table 3** shows alpha (within sample) diversity of the entire cohort, the bacterial richness (the estimated number of taxa) decreased significantly over time (P=0.01). Other measures of diversity did not change across time.

**Table 3:** Microbiome diversity measurements across the three timepoints.

	Time points			
Indices	Beginning ^a^ N=31	Middle ^b^ N=79	End ^c^ N=59	P-value
Shannon Index - Median (IQR)	1.32 (1.14 – 1.55)	1.18 (0.66 – 1.58)	1.10 (0.54 – 1.44)	0.13
Simpson index - Median (IQR)	0.35 (0.31 – 0.50)	0.41 (0.27 – 0.74)	0.47 (0.30 – 0.79)	0.25
Microbiota richness - Median (IQR)	12 (11 – 14)	11 (10 – 13)	11 (9 – 12)	0.01
Pielou evenness - Median (IQR)	0.55 (0.43 – 0.60)	0.49 (0.30 – 0.65)	0.50 (0.22 – 0.60)	0.62

**Statistical significant value at P<.05. All P-values are from Kruskal-Wallis Test.

a Beginning timepoints included those samples obtained before initiation of intravesical therapy.

b Middle timepoints included samples obtained after at least one instillation of intravesical therapy but prior to last instillation of intravesical therapy.

c End timepoints included samples obtained prior to final instillation of intravesical therapy or after all intravesical therapy treatments were completed.

**Figure 1:**
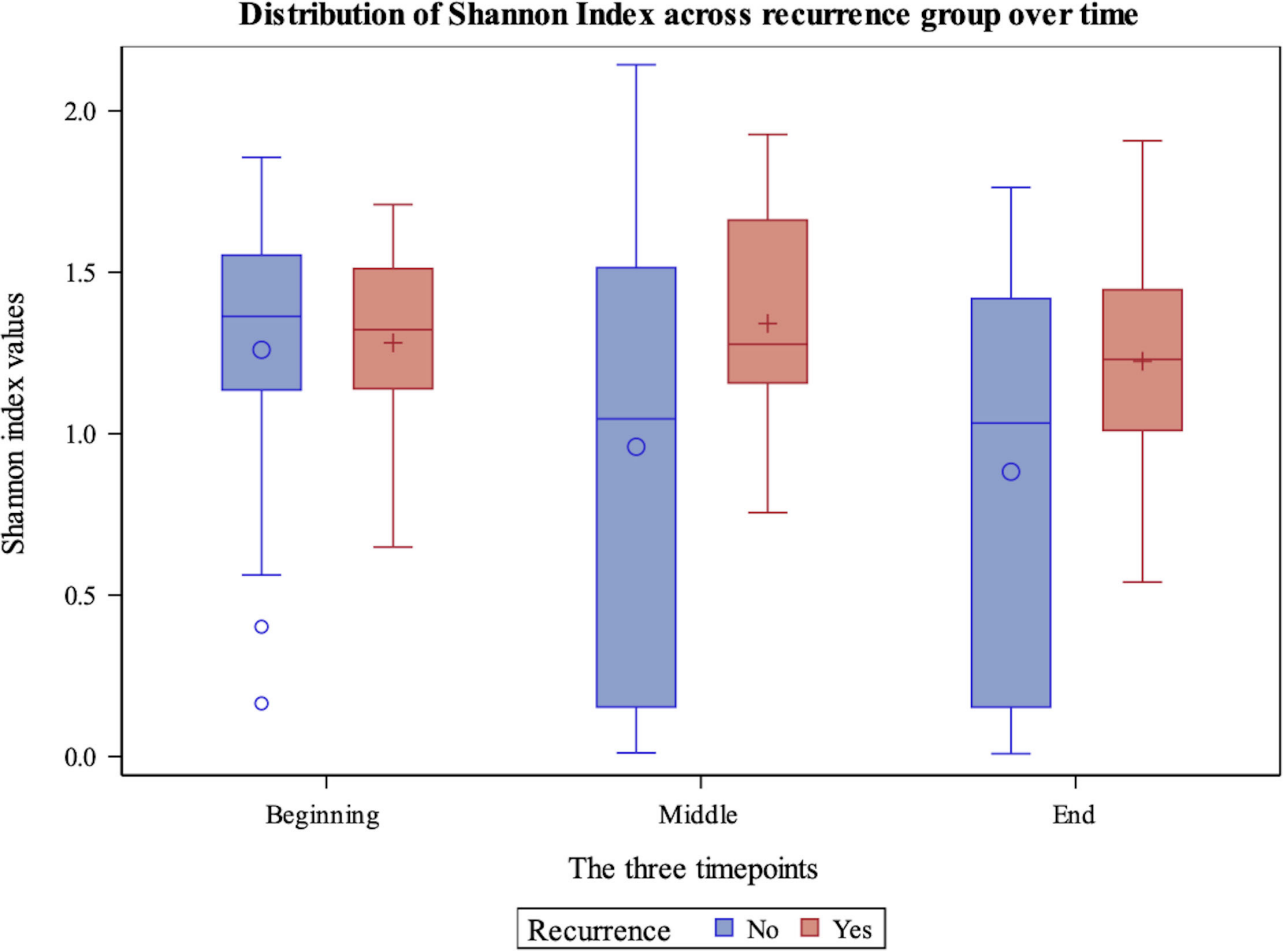
Box plot demonstrating Shannon Diversity Index measures for non-recurrence and recurrence patients within the cohort across beginning, middle and end timepoints. The y-axis displays the value of the Shannon Diversity Index. The x-axis indicates timepoint.

**Table 4** shows the comparison of alpha diversity measures from 16S rRNA gene sequencing between patients who experienced recurrence versus those who did not. The Shannon and Simpson indices differed significantly (P=0.02 and 0.01 respectively) with patients who recurred having more diverse bladder microbiomes than those who did not recur. These differences were likely due to increased evenness in the group that recurred (P=0.01) as there was no difference in richness. These relationships also can be seen over the duration of treatment. The Shannon index shows that patients whose overall alpha diversity decreased during therapy were more likely to fall within the non-recurrence cohort, while there was no difference in recurrence propensity in patients with more diversity (**Figure 1**). This trend was also observed when alpha diversity was calculated by either the Simpson or Pielou indices, but not richness (**Supplemental Figures 2**-**4**).

**Table 4:** Diversity measures by recurrence.

	Overall	Recurrence	No Recurrence	p value
Shannon Index ~ Median (IQR)	1.20 (0.73)	1.23 (0.49)	1.12 (1.17)	0.02
Simpson Index ~ Median (IQR)	0.41 (0.40)	0.35 (0.21)	0.46 (0.59)	0.01
Microbiota richness ~ Median (IQR)	11 (3)	11 (2)	11 (3)	0.56
Pielou evenness ~ Median (IQR)	0.51 (0.29)	0.54 (0.16)	0.49 (0.47)	0.01

IQR, Interquartile Range.

P values from Wilcoxon rank sum tests (medians).

Finally, **Table 5** shows the most common bacterial genera detected in the recurrence and non-recurrence groups *via* 16S rRNA gene sequencing. Relative abundance of most species was similar between the two groups with some notable exceptions. The relative abundance of the genus *Aerococcus* was significantly higher in the recurrence group (P<0.01). In contrast, the relative abundance of the genera *Escherichia/Shigella* and *Ureaplasma* was significantly higher in the non-recurrence group (P=0.05 and 0.01, respectively).

**Table 5:** Relative abundance of genera.

	Recurrence	Non-recurrence	P-value
	(N=49)	(N=120)	
Genus			
*Enterococcus*	0.82 (0.39)	0.86 (0.35)	0.5
*Citrobacter*	0.24 (0.43)	0.32 (0.47)	0.36
*Escherichia/Shigella*	0.51 (0.51)	0.68 (0.47)	0.05
*Lactobacillus*	0.63 (0.48)	0.75 (0.43)	0.13
*Pseudomonas*	0.78 (0.42)	0.79 (0.41)	0.82
*Corynebacterium*	0.86 (0.35)	0.88 (0.32)	0.64
*Klebsiella*	0.49 (0.51)	0.38 (0.49)	0.17
*Staphylococcus*	0.94 (0.24)	0.93 (0.26)	0.76
*Peptoniphilus*	0.90 (0.31)	0.86 (0.35)	0.49
*Streptococcus*	0.71 (0.46)	0.68 (0.47)	0.7
*Actinotignum*	0.53 (0.50)	0.41 (0.49)	0.15
*Aerococcus*	0.65 (0.48)	0.36 (0.48)	<0.01
*Anaerococcus*	0.84 (0.37)	0.75 (0.43)	0.22
*Gardnerella*	0.29 (0.46)	0.36 (0.48)	0.37
*Bifidobacterium*	0.22 (0.42)	0.34 (0.48)	0.14
*Ureaplasma*	0.10 (0.31)	0.29 (0.46)	0.01
*Campylobacter*	0.45 (0.50)	0.44 (0.50)	0.93
*Mycobacterium*	0.22 (0.42)	0.35 (0.48)	0.11
Other	1 (0)	1 (0)	1.00

Values are recorded as Mean (std).

P values from Wilcoxon rank sum tests (medians).

**Figure 1** demonstrates Shannon diversity measures between the recurrence and non-recurrence groups with regards to 16S rRNA gene sequencing results. Patients with decreased levels of alpha diversity were more likely to fall within the non-recurrence cohort, while there was no difference in recurrence propensity in patients with more alpha diversity.

## Discussion

It is not known how BCG or intravesical gemcitabine affect the urinary microbiome in patients with NMIBC (Fuge et al., 2015). Thus, we aimed to determine whether intravesical therapy alters the bladder microbiome in patients undergoing treatment for NMIBC. We also investigated bacterial trends in patients who recurred after intravesical therapy as compared to those who did not.

We observed a cohort-wide statistically significant decrease in bacterial richness throughout treatment. This decrease in bacterial richness may indicate a BCG-mediated immune response against bladder microbiome inhabitants, as BCG is known to elicit an immune response within the bladder (Lim et al., 2021). It should be noted that this decrease in richness during intravesical therapy was not universal across the cohort with 13 out of the 29 patients either exhibiting no change or an increase in richness.

After performing a sub-analysis of our cohort and separating those who recurred after intravesical therapy versus those who did not recur, we made several notable findings. First, when we compared alpha diversity measures between the two groups, it was noted that individuals within the non-recurrence group exhibit less alpha diversity. Of the individuals within the overall cohort that exhibited high levels of alpha diversity, there was no difference between the two groups. Existing data on bacterial diversity in recurrent NMIBC have been mixed. Bucevic Popovic et al. reported no difference in alpha diversity measures between 12 patients with NMIBC compared to 11 healthy controls (Bucevic Popovic et al., 2018). In contrast, Zeng et al. reported higher species diversity in 62 patients with recurrent NMIBC compared to 19 healthy controls (Zeng et al., 2020). If supported by larger controlled studies, our findings with regards to differences in diversity measures may provide future means by which to risk-stratify patients prior to adjuvant intravesical therapy for NMIBC. Second, we observed bacterial trends within our patient cohort that may be associated with BCG treatment efficacy. We found that the genus *Aerococcus* was present at higher levels in patients who experienced a recurrence after intravesical therapy compared to those patients who did not recur (P<0.01). We also noted that the genera *Escherichia/Shigella* and *Ureaplasma* were present at higher levels at all timepoints in non-recurrent patients compared to the cohort of patients that recurred over this timeframe (P= 0.05 and 0.01, respectively). These findings lend support to the possible interplay between the bladder microbiome and response to intravesical therapy in the context of bladder cancer. If the association of these bacteria with recurrence propensity is proven by larger, controlled studies, this may prove to be an additional method with which to risk-stratify patients and provide a target for treatment. We did not control for other factors known to impact response to intravesical therapy such as presence of high-risk mutations (p53 loss or mutation) (Knowles and Hurst, 2015), so it is also possible these findings may be associated with tumor biology alone.

Knorr et al. have published on their findings investigating potential correlation between the baseline microbiome composition and BCG response (Knorr et al., 2021). No significant richness differences in the pre-BCG microbiome were observed between responders and non-responders. One notably significant finding in their study was the enrichment of *Corynebacterium* in BCG responders. The theory proposed by the authors is that *Corynebacterium* and *M. bovis* share taxonomic similarities which may suggest the use of *Corynebacterium* as a pre-treatment marker for favorable BCG response. More recently Knorr et al. published data investigating urinary microbiome trends from bladder tumor tissue in a larger series of 47 patients undergoing BCG treatment for bladder cancer (Knorr et al., 2022). This study found significant enrichment of *Lactobacillus* in BCG responders and significant enrichment of *Corynebacterium* in BCG non-responders, in contrast to their previous findings. Thus, microbe-specific BCG response predictors remain uncertain.

To our knowledge, only one other study investigating the longitudinal impact of intravesical therapy on the urinary microbiome exists. Sweis and co-workers investigated the bacterial composition of 31 patients undergoing BCG treatment for NMIBC *via* 16S rRNA gene sequence analysis. They observed a significant difference in bacterial composition by distance matrix computation between patients with and without recurrence (Bonferroni-corrected P=0.017) (Sweis et al., 2019). They also found the abundance of the phylum Proteobacteria was higher in patients with recurrence (P=0.035), with stronger differences observed for specific classes such as Gammaproteobacteria (P=0.0025). This study also found that certain members of the phylum Firmicutes, such as Lactobacillales, were more abundant in patients who did not recur (P=0.049). When comparing our findings to those of Sweis and co-workers, there are notable differences. First, the bacterial taxa found to be associated with recurrence versus those associated with favorable intravesical therapy response were quite different. The phylum Proteobacteria phylum contains the genera *Escherichia/Shigella* and *Ureaplasma*. Our study showed these bacteria to be detected at statistically significant higher abundance in patients who had a favorable response to intravesical therapy, whereas Sweis and team found more abundance of Proteobacteria members in patients who recurred after BCG. Another difference between the findings of the two studies is the role of Firmicutes in the response to intravesical therapy. Our study found that one Firmicute genus, *Aerococcus*, was detected at higher abundance in those patients who recurred after intravesical therapy. Sweis and team found that increased Firmicute abundance, especially the genus *Lactobacillus*, was associated with favorable BCG response. Both studies found differences in the bacterial compositions between patients with and without recurrence after intravesical therapy; however, it is not known which diversity statistics were performed by Sweis and co-workers, if any. While our results do not agree with the findings of Sweis and team, both studies are small, demonstrating the need for larger, controlled studies. In addition, Sweis and team provided scant demographic data with regards to their cohort. It is possible that our cohorts are vastly different in nature, which could explain the stark differences in findings with regards to the effect of intravesical therapy on the bladder microbiome.

### Strengths and limitations

One major strength of our study is the use of sterile catheterization for urine specimen collection. There is an abundance of data to suggest that voided urine specimens may contain bacteria not only from the bladder, but also from the urethra, the periurethral skin and/or the labial skin. Transurethral catheterization (TUC) and suprapubic aspiration (SPA) have been shown to be the best methods by which to obtain urine most representative of the bladder microbiota (Wolfe et al., 2012). However, due to the invasiveness of SPA, TUC has been the recommended method by which to study the bladder microbiota in research. By obtaining a catheterized urine specimen, we were able to interpret our data as a reliable representation of bladder microbiome composition.

Our study is not without limitations. First, we once again acknowledge the small sample size of our study. However, even within this small cohort of patients, we were able to make statistically significant conclusions regarding the impact of intravesical therapy on the bladder microbiome. We were also able to note possible bacterial trends associated with recurrence versus favorable response after intravesical therapy. Larger cohort studies are required to validate this information, but our study does provide promising future directions.

We also acknowledge that the sub-analysis comparing patients who recurred versus those who had favorable response to intravesical therapy suffers from a difference in demographics. Specifically, there were racial disparities between the two groups that may confound results and prohibit conclusions explicit to the bladder microbiome and efficacy of BCG treatment. In addition, there is a significant difference in the number of patients who received BCG and gemcitabine. This is due to the national BCG shortage experienced during a portion of our accrual period. BCG and gemcitabine have notably different cytotoxic mechanisms of action that may impact the bladder microbiota differently. Our goal in this study was to determine if intravesical therapies alter the bladder microbiota in patients with NMIBC which we were able to do. Future studies may focus on each intravesical bladder cancer treatment agent to determine the agent-specific changes that occur in the bladder microbiota.

A final acknowledgment of limitation within our study is the use of urine as a surrogate for the true urothelial microbiota. In a 2021 study investigating the presence of bacterial biofilms on bladder cancer tissue, Nadler et al. described dense bacterial bladder tumor tissue aggregates in two out of ten patients in their cohort (Nadler et al., 2021). This promising pilot study suggests a potential interplay between bladder tumor tissue and its associated biofilm, although further, larger studies are necessary to potentiate this significance. One notable finding in their study is that the two patients found to have dense biofilms adherent to their bladder tumors, neither of them had a history of positive urine cultures. This begs the question of whether urine is an accurate surrogate for the urothelial tissue microbiome.

A 2023 study by Wolfe et al. compared the microbiota of women with interstitial cystitis to healthy controls (Wolfe et al., 2023). As part of this study, catheterized urine and urothelial biopsy tissue were analyzed using 16S rRNA gene sequencing and compared. Urothelial tissue was also imaged to confirm 16S rRNA gene sequencing findings. This study concluded that the urothelial and urinary microbiota are similar but demonstrate distinct differences in genera abundance. These findings lend support to using urine as a surrogate for the urothelial microbiota, but larger studies are required to fully investigate this.

## Conclusion

Our study demonstrates a cohort-wide decrease in microbiome richness during BCG or intravesical therapy for NMIBC. We also found that non-recurrence patients had higher levels of *Escherichia/Shigella* and *Ureaplasma*, while patients who recurred after BCG or intravesical therapy had higher levels of *Aerococcus*. Finally, individuals within the non-recurrence group tended to become less diverse over the course of treatment. These findings lend insight into future directions of investigation that may provide meaningful diagnostic and treatment information for patients undergoing intravesical therapy for NMIBC.

## Data availability statement

The datasets presented in this study can be found in online repositories. The names of the repository/repositories and accession number(s) can be found below:. https://www.ncbi.nlm.nih.gov/, PRJNA909316.

## Ethics statement

The studies involving human/animal participants were reviewed and approved by the Institutional Review Board at Loyola University. The patients/participants provided their written informed consent to participate in this study.

## Author contributions

Study concept and design: RD, CD, PB, TH, GG, MQ, AG, RF, AW. Data acquisition: RD, CD, AW. Data analysis: RD, CD, SD, CJ, AW. Drafting of manuscript: CJ, AW. All authors contributed to the article and approved the submitted version.

## Supplementary material

Supplementary Figure 1 Prevalence of the most abundant genera for the beginning, middle and end timepoints. Note that the middle contains data from multiple timepoints whereas the beginning and end are from single timepoints.

Supplementary Figure 2 Box plot demonstrating Simpson Diversity Index measures for non-recurrence and recurrence patients within the cohort across beginning, middle and end timepoints. The y-axis displays the value of the Simpson Diversity Index. The x-axis indicates timepoint.

Supplementary Figure 3 Box plot demonstrating richness measures for non-recurrence and recurrence patients within the cohort across beginning, middle and end timepoints. The y-axis displays the richness values. The x-axis indicates timepoint.

Supplementary Figure 4 Box plot demonstrating evenness measures for non-recurrence and recurrence patients within the cohort across beginning, middle and end timepoints. The y-axis displays evenness value. The x-axis indicates timepoint.

Supplementary Table 1 Detailed sample processing, preparation, bioinformatic, and analytic methods for 16S rRNA gene sequencing.

Supplementary Table 2 16S rDNA V4 Amplicon Library Primers
