# Supplementary material for: Impact of intravesical Bacillus Calmette-Guérin and chemotherapy on the bladder microbiome in patients with non-muscle invasive bladder cancer
Source: Front Cell Infect Microbiol. 2023 Apr 5;13:1125809. doi: 10.3389/fcimb.2023.1125809 (PMC10114608; doi:10.3389/fcimb.2023.1125809)
Supplement: Supplementary Figure 1 — Prevalence of the most abundant genera for the beginning, middle and end timepoints. Note that the middle contains data from multiple timepoints whereas the beginning and end are from single timepoints. [file DataSheet_1.docx]

# Impact of Intravesical Bacillus Calmette-Guérin and Chemotherapy on

# the Bladder Microbiome in Patients With Non-Muscle Invasive Bladder Cancer

Christopher James, MD^1^; Kayeromi Gomez^2^ ; Shalin Desai, MD^1^; Hiten Patel, MD^1^; Goran Rac, MD^1^; Chirag P. Doshi, MD^1^; Ryan Dornbier, MD^1^; Petar Bajic, MD^1^; Gopal Gupta, MD^1^; Marcus Quek, MD^1^; Alex Gorbonos, MD^1^; Robert Flanigan, MD^1^; Alan J. Wolfe, PhD^3^

# SUPPLEMENTAL INFORMATION


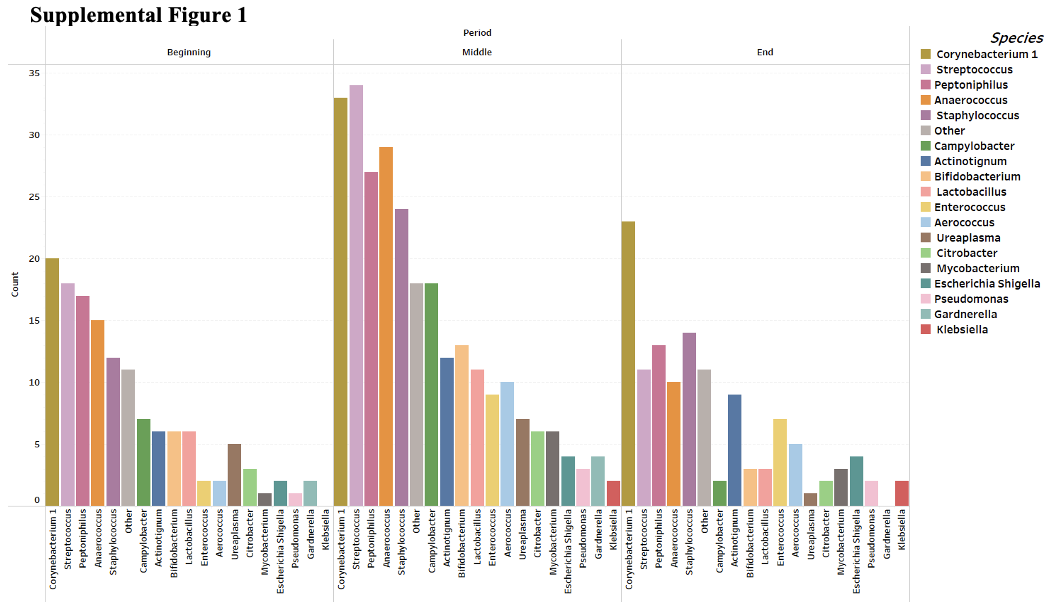


**Supplemental Figure 2**

**Supplemental Figure 3**

**Supplemental Figure 4**
