# Supplementary material for: Impact of intravesical Bacillus Calmette-Guérin and chemotherapy on the bladder microbiome in patients with non-muscle invasive bladder cancer
Source: Front Cell Infect Microbiol. 2023 Apr 5;13:1125809. doi: 10.3389/fcimb.2023.1125809 (PMC10114608; doi:10.3389/fcimb.2023.1125809)
Supplement: Supplementary Table 1 — Detailed sample processing, preparation, bioinformatic, and analytic methods for 16S rRNA gene sequencing. [file Table_1.docx]

**Supplemental Table 1: 16S rDNA V4 Amplicon Library Primers**

| **Primer** | **Primer sequence (5’ to 3’)** |
| --- | --- |
| **Primary PCR primers** | |
| Modified 515F | TCGTCGGCAGCGTCAGATGTGTATAAGAGACAGGTGCCAGCMGCCGCGGTAA |
| Modified 806R | GTCTCGTGGGCTCGGAGATGTGTATAAGAGACAGGGACTACHVGGGTWTCTAAT |
