# Supplementary material for: Impact of intravesical Bacillus Calmette-Guérin and chemotherapy on the bladder microbiome in patients with non-muscle invasive bladder cancer
Source: Front Cell Infect Microbiol. 2023 Apr 5;13:1125809. doi: 10.3389/fcimb.2023.1125809 (PMC10114608; doi:10.3389/fcimb.2023.1125809)
Supplement: Supplementary Table 2 — 16S rDNA V4 Amplicon Library Primers [file Table_2.docx]

Supplemental Information:

**Supplemental Table 2:** Detailed sample processing, preparation, bioinformatic, and analytic methods for 16S rRNA gene sequencing

| Method | Study-specific information | References |
| --- | --- | --- |
| Initial urine storage and transport | Urine was placed into 50mL conical tubes pre-loaded with 10% Assay Assure™ (Thermo Fisher Scientific, Waltham, MA); stored at -80°C for future batched sequencing. | Specimen transport & processing^1^ |
| Laboratory processing & creation of cell pellets | Centrifuged at 13,500 rpm for 10 minutes after thawing to 4°C to pellet the cells. Cells re-suspended in 200 microliters of filter-sterilized buffer consisting of 20 mM Tris-Cl (pH 8), 2mM EDTA, 1.2% Triton X-100, and 20 micrograms/ml lysozyme supplemented with 20 microliters of filter-sterilized mutanolysin (5,000 U/ml; Sigma-Aldrich, St. Louis, MO). Mixture was incubated at 37°C for 1 hour. |  |
| DNA extraction | Thawed cell pellets subjected to the cultured cell protocol of the Qiagen DNeasy Blood and Tissue Kit (Valencia, CA, product code # 69506). Addition of RNase step (optional). Genomic DNA was eluted into 50 μL of buffer AE (pH 8.0) and stored at -20°C | DNA extraction for urobiome studies^2^ |
| Controls & dilution series of mock microbial community | Negative control: no urine or swab suspension phosphate-buffered saline  Positive control: Mock microbial community (Zymo Research, catalog # D6300)  Dilution series: Diluted reaction mixtures 1:50 and amplified them for 10 cycles  All positive and negative controls were subjected to the same DNA extraction steps using the same reagents as the study samples. | Controlling for contaminants in low biomass samples^3^ |
| Library preparation for 16S rRNA gene sequencing | PCR amplification of the V4 hypervariable region of the 16S rRNA gene using the forward primer 515 and reverse primer 806R.  Equimolar PCR products from all samples were pooled prior to sequencing. | Earth Microbiome Project^4,5^ |
| Sequencing | Illumina MiSeq instrument configured for 250 base-pair paired-end |  |
| Bioinformatic processing & taxonomic identification | Raw reads were quality trimmed using Cutadapt (v.2.7) to remove adaptors from both ends. Trimmed reads processed using DADA2 package (v 1.14.1)   - Including quality control, error rate calculation, dereplication, and chimera removal to generate amplicon sequence variant (ASV) tables - SILVA reference database (v 132) used to assign taxonomy to the sequences in the ASV table | Processing with DADA2^6,7^ |
| Bioinformatic identification of contaminants | Decontam (v 1.2.1) was used to identify potential contaminant ASVs using the frequency method and a threshold of 0.5, which was chosen after evaluating the contaminant removal of the mock microbial dilution series. | Apply decontam to remove contaminants^8^ |
| Bioinformatic filtering, decontamination, and normalization | Additional filtering process was used to confidently analyze only ASVs that were not contaminants: 1) ASVs whose reads did not exceed 5 times the maximum number of reads in extraction and negative controls were removed  2) Samples with less than 1000 reads were discarded from downstream analysis  After decontamination, taxonomic identities for the remaining ASVs were obtained using BLCA | Use BLCA to obtain taxonomic identities for remaining ASVs^9^ |
| Statistical Analyses | All calculations and data analysis performed using the SAS statistical software (v 9.4)  1) Kruskal Wallis tests were also used for comparison across the three timepoints (beginning, the middle and end)  2) Richness was calculated using the number (counts) of unique species 3) The distribution of microbial species within samples (evenness) was computed with the Pielou index  4) Combined interactions were calculated with the Shannon index (richness and evenness) and Simpson index (richness and species abundance). | SAS Statistical software^10^  Kruskal Wallis test^11^  Pielou, Shannon and Simpson Indices^12^ |

*The complete urine volume was collected and evenly divided. For those with large volumes, anything in excess of 200mL was discarded.

**Supplemental References:**

1. Fok CS, Gao X, Lin H, Thomas-White KJ, Mueller ER, Wolfe AJ, Dong Q, Brubaker L. Urinary symptoms are associated with certain urinary microbes in urogynecologic surgical patients. Int Urogynecol J. 2018 Dec;29(12):1765-1771. doi: 10.1007/s00192-018-3732-1. Epub 2018 Aug 16. PMID: 30116843; PMCID: PMC6830733.
2. Karstens L, Siddiqui NY, Zaza T, et al. Benchmarking DNA isolation kits used in analyses of the urinary microbiome. *bioRxiv.* 2020.
3. Karstens L, Asquith M, Davin S, et al. Controlling for Contaminants in Low-Biomass 16S rRNA Gene Sequencing Experiments. *mSystems.* 2019;4(4).
4. Thompson LR, Sanders JG, McDonald D, et al. A communal catalogue reveals Earth's multiscale microbial diversity. *Nature.* 2017;551(7681):457-463.
5. Earth Microbiome Project. <https://www.earthmicrobiome.org/>. Updated 2021. Accessed 2/22/2021.
6. Callahan BJ, McMurdie PJ, Rosen MJ, et al. DADA2: High-resolution sample inference from Illumina amplicon data. *Nat Methods.* 2016;13(7):581-583.
7. Callahan BJ, McMurdie PJ, Holmes SP. Exact sequence variants should replace operational taxonomic units in marker-gene data analysis. *ISME J.* 2017;11(12):2639-2643.
8. Davis NM, Proctor DM, Holmes SP, et al. Simple statistical identification and removal of contaminant sequences in marker-gene and metagenomics data. *Microbiome.* 2018;6(1):226.
9. Gao X, Lin H, Revanna K, Dong Q: A Bayesian taxonomic classification method for 16S rRNA gene sequences with improved species-level accuracy. *BMC Bioinformatics* 2017, 18(1):247.
10. Rott KW, Lin L, Hodges JS, Siegel L, Shi A, Chen Y, Chu H. Bayesian meta-analysis using SAS PROC BGLIMM. Res Synth Methods. 2021 Nov;12(6):692-700. doi: 10.1002/jrsm.1513. Epub 2021 Jul 21. PMID: 34245227; PMCID: PMC8867920.
11. Gao R, Zhu Y, Kong C, Xia K, Li H, Zhu Y, Zhang X, Liu Y, Zhong H, Yang R, Chen C, Qin N, Qin H. Alterations, Interactions, and Diagnostic Potential of Gut Bacteria and Viruses in Colorectal Cancer. Front Cell Infect Microbiol. 2021 Jul 6;11:657867. doi: 10.3389/fcimb.2021.657867. PMID: 34307189; PMCID: PMC8294192. 12. Anderson MJ. A new method for non-parametric multivariate analysis of variance. *Austral Ecology.* 2001;26(1):32-46.
12. Mouillot, D., Leprêtre, A. A comparison of species diversity estimators. *Res Popul Ecol* **41**, 203–215 (1999). https://doi.org/10.1007/s101440050024
